# Infectious Complications of Pulmonary Sarcoidosis

**DOI:** 10.3390/jcm13020342

**Published:** 2024-01-07

**Authors:** Dominique Valeyre, Jean-François Bernaudin, Michel Brauner, Hilario Nunes, Florence Jeny

**Affiliations:** 1 INSERM-UMR 1272, SMBH Université Sorbonne Paris-Nord, 93009 Bobigny, France; dominique.valeyre@gmail.com (D.V.); jf.bernaudin-univ@orange.fr (J.-F.B.); hilario.nunes@aphp.fr (H.N.); 2 Service de Pneumologie, Groupe Hospitalier Paris Saint Joseph, 75014 Paris, France; 3 Faculty of Medicine, Sorbonne University, 75013 Paris, France; 4 Service de Radiologie, Hôpital Avicenne, 93009 Bobigny, France; michel.brauner@wanadoo.fr; 5 Service de Pneumologie, Hôpital Avicenne, 93009 Bobigny, France

**Keywords:** infection, sarcoidosis, opportunistic infections, mycoses, tuberculosis, COVID-19

## Abstract

In this review, the infectious complications observed in sarcoidosis are considered from a practical point of view to help the clinician not to overlook them in a difficult context, as pulmonary sarcoidosis makes the recognition of superinfections more difficult. An increased incidence of community-acquired pneumonia and of opportunistic pneumonia has been reported, especially in immunosuppressed patients. Pulmonary destructive lesions of advanced sarcoidosis increase the incidence of chronic pulmonary aspergillosis and infection by other agents. Screening and treatment of latent tuberculosis infection are crucial to prevent severe tuberculosis. Severity in COVID-19 appears to be increased by comorbidities rather than by sarcoidosis per se. The diagnosis of infectious complications can be challenging and should be considered as a potential differential diagnosis when the exacerbation of sarcoidosis is suspected. These complications not only increase the need for hospitalizations, but also increase the risk of death. This aspect must be carefully considered when assessing the overall health burden associated with sarcoidosis. The impact of immune dysregulation on infectious risk is unclear except in exceptional cases. In the absence of evidence-based studies on immunosuppressants in the specific context of pulmonary sarcoidosis, it is recommended to apply guidelines used in areas outside sarcoidosis. Preventive measures are essential, beginning with an appropriate use of immunosuppressants and the avoidance of unjustified treatments and doses. This approach should take into account the risk of tuberculosis, especially in highly endemic countries. Additionally, parallel emphasis should be placed on vaccinations, especially against COVID-19.

## 1. Introduction

Pulmonary sarcoidosis is a systemic granulomatous disease primarily affecting the lungs and lymphatic system, with a range of clinical presentations and outcomes spanning from spontaneous remission to progressive, severe respiratory dysfunction [1,2]. The etiology remains unknown [1,2]. While some patients may require no intervention, more than half of patients with sarcoidosis require immunosuppressive therapy, such as glucocorticosteroids, cytotoxic drugs, or biologics, for periods ranging from one to several years [3].

The relationship between infectious agents and sarcoidosis is complex. Although there is a hypothesis that infectious agents may play a role in the pathogenesis of sarcoidosis, this remains speculative. Conversely, infectious diseases can also complicate sarcoidosis, leading to repeated hospitalizations, respiratory exacerbations, and/or death [4]. Infectious complications consist primarily of community-acquired pneumonia (CAP) and immunocompromised host pneumonia (ICHP) associated with immunosuppressive treatment. Parenchymal fibrocystic lung disease may increase susceptibility to superinfections [5,6,7]. Some infectious diseases may be associated with epidemiologic conditions, such as exposure to tuberculosis, histoplasmosis, or the risk of COVID-19. Another important issue is to determine whether sarcoidosis itself may increase the likelihood of infection, independent of immunosuppressants and fibrocystic lung lesions. 

It can be challenging to distinguish an extraneous infection from a true exacerbation of sarcoidosis or another complication, such as left ventricular failure or pulmonary thromboembolism [8,9]. Detecting manifestations of infection on lung imaging can be particularly challenging. In specific cases, the severity of an infection may be more dependent on the underlying advanced pulmonary, cardiac, or neurologic sarcoidosis than on the infection itself. Infections can pose an additional health burden, affecting hospitalizations, care, work disability, quality of life, and mortality [10,11].

This review aims to provide practical insights into the management of infectious complications in sarcoidosis. Dedicated sections will address data obtained from well-conducted epidemiologic studies, reports on immunocompromised host infections, fungal infections, the suprainfections of pulmonary fibrocystic lesions, tuberculosis, COVID-19, and curative and preventive treatments. Additionally, the review will explore the key impaired anti-infectious mechanisms in sarcoidosis, facilitate discussion on these critical topics, and provide future research directions in this field.

## 2. Methods

We searched through PubMed for original articles and reviews, and, in some cases, reports published in the English language after 2020; older publications have also been included according to the importance of the information. Therefore, we used the term “sarcoidosis” in combination with the following terms: “infectious risk”, “hospitalized infections”, “death certificate”, “tuberculosis”, “COVID-19”, “Aspergillosis”, “Pneumocystosis”, “community-acquired pneumonia”, “opportunistic infection”, “vaccination”, “prevention”, and “treatment”. Eventually, articles about B-cell immunity, T Regs and autophagy, and defenses against infectious agents in sarcoidosis were considered. In general, the main goal of this review was to focus on the infectious complications by themselves.

## 3. Well-Conducted Epidemiologic Studies on the Association between Infectious Diseases and Sarcoidosis

Several studies providing insights into the association between infectious diseases and sarcoidosis are based on registry-based studies conducted in Sweden, the US, Taiwan, and France [12,13,14,15,16,17,18]. These studies have focused on (i) infections that occurred prior to the diagnosis of sarcoidosis, (ii) infections that occurred after the diagnosis of sarcoidosis, (iii) the exploration of the relationship between tuberculosis and sarcoidosis, and (iv) the investigation of the impact of infectious diseases as a cause of death in patients with sarcoidosis.

Studies investigating the causal role of infectious diseases in sarcoidosis are warranted, because the “nature” heritability of sarcoidosis is reported to account only for 31% of the causes of sarcoidosis, whereas the “nurture” exposure to occupational, environmental, or infectious airborne contaminants accounts for up to 69% [19]. An association has been identified with a history of infectious disease, including upper respiratory and ocular infections, at least three years prior to the sarcoidosis diagnosis. This association is linked to a 25% increased likelihood of being diagnosed with sarcoidosis [12]. A causal role for infections must be considered with caution. Assuming that 1 in 10 infections occurs in preclinical sarcoidosis, with a possible underlying immune disorder, the increased risk is substantially mitigated. In addition, this study showed that, if there is indeed a true causal role of infection in the development of sarcoidosis, its quantitative impact appears to be relatively weak. Taking into account the risk of infection postdiagnosis of sarcoidosis, a study conducted between 2006 and 2013, before the COVID-19 outbreak, found that sarcoidosis was associated with a 1.8-fold increased risk of first serious infection, i.e., hospitalization with an ICD code for an infectious disease, compared to the general population. Notably, this increased risk was most significant in the first two years after the diagnosis of sarcoidosis [13]. The hazard ratio for serious infection was three times higher in individuals who received immunosuppressants after diagnosis. Even untreated sarcoidosis patients had a 50% increased risk of serious infection compared to controls. Interestingly, the risk was much lower in sarcoidosis than in lupus or COPD. The rate of recurrent serious infections was also doubled in sarcoidosis patients.

Hospitalization for multiple serious infections occurred with unusual frequency in sarcoidosis patients. In another US study, using the Rochester Epidemiology Project, similar results were obtained by comparing the risk of hospitalized infections between sarcoidosis patients and controls [14]. The HR was 1.73 in untreated sarcoidosis patients; the risk was higher (HR = 3.3) in patients on low doses of glucocorticosteroids, and there was a higher risk (HR = 4.48) when glucocorticosteroids were administered at doses above 10 mg/d. Notably, a study by Larsson et al., using the Swedish National Patient Register, showed a higher incidence of influenza or pneumonia in sarcoidosis patients compared to controls (HR 2.98) [17].

Methotrexate and azathioprine are immunosuppressants used as a second-line treatment for sarcoidosis [15]. Within 6 months of treatment, methotrexate was associated with a 43% reduced risk of infectious disease compared to azathioprine, with a respective risk of 6.8% versus 12% [15].

A nationwide study of the Taiwan National Health Insurance Database from 2000 to 2015 examined the risk of sarcoidosis after a tuberculosis diagnosis compared to a control population without tuberculosis. Conversely, the risk of tuberculosis based on a prior diagnosis of sarcoidosis was also examined compared to a control population without sarcoidosis [16]. In Taiwan, where the incidence of tuberculosis was still high during the study (57 p 100,000 in 2012), the risk of sarcoidosis was 8.09 times higher in patients with tuberculosis than in controls without tuberculosis, suggesting that a history of tuberculosis is a risk factor for the development of sarcoidosis. A latent onset of sarcoidosis, emerging long after the tuberculosis had been treated, was suggested. Interestingly, the risk of sarcoidosis was higher in extrapulmonary tuberculosis than in pulmonary tuberculosis [16]. Conversely, the risk of tuberculosis after a diagnosis of sarcoidosis was higher than in controls without sarcoidosis (HR 1.85). Given the common presentation of tuberculosis and sarcoidosis, and the possible confusion between the two diagnoses, the interpretation of these results must be discussed (see the Section 12).

Thanks to an analysis by the French Epidemiological Center for Medical Causes of Death from 2002 to 2011, it was possible to calculate age- and sex-adjusted observed/expected ratios in sarcoidosis patients compared to the general population for the underlying cause of death when sarcoidosis was listed as a nonunderlying cause of death. The analysis showed an increased ratio for infection in sarcoidosis [18]. Infection was the underlying cause of death in 11.7% of women and 8.6% of men with sarcoidosis.

## 4. Immunocompromised Host Infections

In this section, we will focus on the so-called immunocompromised host pneumonia (ICHP) and briefly address extrapulmonary sites of infection. ICHP was recently defined by the American Thoracic Society workshop as an infectious pneumonia affecting an individual with a quantitative or functional host immune defense disorder [20]. Chronic immunosuppression is a major risk for sarcoidosis patients. This is most frequently attributed to the use of immunosuppressants, which are administered to approximately half of all patients. These treatments include corticosteroids, cytotoxic agents, and biologics. In addition, solid organ transplantation, including lung, heart, liver, and kidney transplantation, may also contribute to immunosuppression. 

Most of sarcoidosis treatments impair the function of various immune cell types, including macrophages and T-lymphocytes. This may increase the risk of developing *Pneumocystis* pneumonia, invasive aspergillosis, as well as typical CAP. 

It is important to emphasize the need to systematically exclude granulomatosis-associated common variable immunodeficiency disorder as a differential diagnosis, which often results in frequent suprainfections. This can be done by measuring and looking for hypogammaglobulinemia with decreased serum levels of immunoglobulin G (IgG) and immunoglobulin A (IgA) or immunoglobulin M (IgM) [2]. 

### 4.1. Risk of Immunosuppressants

Corticosteroids are recommended as first-line treatment option and may compromise immunity when administered at a dose of at least 20 mg/day of a prednisone equivalent for at least two weeks or 10 mg/day for a longer period. Methotrexate is the preferred second-line treatment, while TNFα blockers, such as infliximab or adalimumab, are preferred as the third-line treatment. TNFα blockers significantly increase susceptibility to *Mycobacterium tuberculosis* and endemic fungal infections [21]. Therefore, individuals from regions with a high incidence of tuberculosis, or those with latent tuberculosis, are at increased risk of developing tuberculosis disease in the absence of preventive measures. The same is true, to a lesser extent, for histoplasmosis. Rarely used, JAK inhibitors and IL6—possibly used in the treatment of multiresistant sarcoidosis—are associated with an increased risk of tuberculosis.

Corticosteroids have been studied in randomized controlled trials for rheumatoid arthritis, but these trials were not well-powered to assess the risk of infection. These studies did not show an increased risk of infection associated with corticosteroids [22]. Nevertheless, observational studies have demonstrated a dose- and duration-dependent increased risk of infections, especially tuberculosis, pneumocystosis, and herpes [22]. In a retrospective study, Vorselaars et al. compared the incidence of infections based on antibiotic use or hospital admissions and found a higher incidence of infections with azathioprine (36.8%) compared to methotrexate (18.1%). This finding is consistent with Rossides’ epidemiologic study in Sweden [15,23]. Several studies have evaluated sarcoidosis patients treated with TNFα blockers, either alone or in combination with other immunosuppressants [24,25]. In Jamilloux’s cohort of 132 patients, one-third of patients experienced infections, such as pneumonia, recurrent urinary tract infections, and bacterial sepsis, often requiring hospitalization and the withdrawal of sarcoidosis treatment [24]. 

This was often followed by a relapse of sarcoidosis. Patients experienced legionellosis, invasive aspergillosis, pneumocystosis, primary cytomegalovirus infection, cryptococcosis, hepatitis B reactivation, and nontuberculous mycobacterial infection, which occurred in one case each. No case of tuberculosis was reported, probably due to recommended preventive measures [24]. Of note, the Heidelberg study, with a median follow-up of 45 months and 46 patients with skin lesions, categorized patients into those receiving treatment for the skin (*n* = 21) or those receiving treatment for an extradermatologic reason (*n* = 25), resulting in a notable finding [25]. There was a significant contrast in infection rates between the two groups, with rates of 9.5% and 48%, respectively. This suggests that the use of TNFα blockers, together with more corticosteroids and cytotoxic drugs in the second group, increased the risk of infection compared to the use of TNFα blockers alone, which were more frequently used in the first group [25]. 

### 4.2. Pulmonary and Extrapulmonary Opportunistic Infections in Sarcoidosis

A study conducted in Rennes, France, showed evidence of a variable risk of *Pneumocystis* pneumonia in non-HIV patients depending on the underlying disease. Among these diseases, sarcoidosis had one of the lowest risks, with fewer than 5 cases per 100,000 patient-years, in contrast to diseases with a high risk, exceeding 70 cases per 100,000 patient-years, such as vasculitis [26].

Interestingly, the incidence of invasive aspergillosis has been reported in rare cases of sarcoidosis [7,24]. 

In a study of 234 patients with neurosarcoidosis receiving immunosuppressive therapies, 7.2% developed treatment-related secondary infections, resulting in three deaths from sepsis [27]. The reporting of infections in this article is critical, as it could potentially lead to significant diagnostic errors, particularly when dealing with sarcoidosis extrapulmonary localizations, and may lead to harmful drug-prescribing. It also raises questions about a defective immunity in some patients with sarcoidosis. According to a Mayo Clinic study, sarcoidosis was the cause of up to 9% of non-HIV-related cases of progressive multifocal leukoencephalopathy (PML), a disease caused by the JC virus (JCV) [28]. The JCV, along with immunosuppression, plays a critical role in the development of PML [28,29]. While corticosteroid therapy, a well-established cause of immunosuppression leading to PML, was present in most sarcoidosis patients, PML has also been observed in several sarcoidosis patients without any immunosuppressive therapy or comorbidity prior to therapy, with PML sometimes being the primary presenting manifestation of sarcoidosis [29]. In PML, brain MRI findings typically differ from those of central nervous system sarcoidosis, with asymmetric subcortical white-matter lesions that are hypointense on T1, hyperintense on T2, and nonenhancing, with the rare exception of contrast enhancement versus contrast-enhancing lesions in the meninges and/or parenchyma. While the cerebrospinal fluid (CSF) cell counts and biochemistry are typically normal in PML, they are more often abnormal in central nervous system sarcoidosis. Therefore, it is important to consider the possibility of PML when the diagnosis of neurosarcoidosis is uncertain, especially in the presence of atypical findings, such as MRI and CSF results. PCR testing is essential for the diagnosis of JCV, with a sensitivity rate of 72–92%. If uncertainty remains, a brain biopsy may be recommended. The prognosis is typically poor, and treatment requires the reversal of immunosuppression. 

In addition, Cryptococcosis is a potential opportunistic infection seen in cases of sarcoidosis. Patients, representing 2.9% of HIV-negative cryptococcosis cases recorded in France [30], manifested the infection during the treatment of sarcoidosis with corticosteroids. However, similar to PML, one-third of the patients experienced cryptococcosis as a revealing manifestation of sarcoidosis, leading to its diagnosis. Organs affected by cryptococcosis included the central nervous system (72%), skin or soft tissue (22%), bones or joints (17%), and liver (11%). Although it is possible, lung infection is very rare. The CSF investigation is highly sensitive, using an India ink preparation, CSF culture, and/or CSF antigen. Patients had a positive prognosis after an antifungal treatment. Interestingly, routine evaluations of immune defenses in both PML and cryptococcosis patients without corticosteroid therapy showed no impairment [30]. 

Herpes zoster may also be responsible for suprainfections, mainly at the ocular level, in patients treated with systemic or topical corticosteroids [31].

## 5. Community-Acquired Pneumonias

Using the National Board of Health of Care and Welfare in Sweden, Larsson demonstrated the higher rate of influenza and pneumonia in sarcoidosis patients compared to controls [17]. *Pneumococcus pneumoniae* is the most common bacterium isolated from adult patients with community-acquired pneumonia. Immunocompromised status increases the prevalence of pneumococcal disease in patients receiving corticosteroids and other immunosuppressive therapies [32,33]. However, there are no available studies in the literature comparing bacterial strains causing pneumonia among sarcoidosis patients and controls.

## 6. Fungal Infections

Several fungal infections have been reported in patients with sarcoidosis. In a US study by Baughman et al. [34] of 753 patients, 0.9% were found to have fungal infections. These included *Histoplasma capsulatum* and *Blastomyces dermatitidis*, both affecting the lungs, and one case of *Cryptococcus neoformans*, leading to meningitis. Diagnosis was most often confirmed by bronchoscopy or lung biopsy, with bone marrow and CSF used in two cases. Histoplasmosis is a fungal infection that is endemic in the United States and some other regions, but not in Europe, except Italy [35]. All documented cases to date have involved patients receiving immunosuppressive treatments, such as corticosteroids with or without methotrexate, and have been successfully treated with antifungal agents after the taper of immunosuppressive medications [34].

On the other hand, pulmonary aspergillosis, which is a widespread infection, has a prevalence of approximately three million cases [36]. Invasive aspergillosis occurs in severely immunocompromised individuals, while chronic pulmonary aspergillosis is commonly associated with tuberculosis, COPD, and sarcoidosis [36]. According to the classification of the European Society of Clinical Microbiology and Infectious Diseases and the European Respiratory Society, chronic cavitary pulmonary aspergillosis is the predominant manifestation of sarcoidosis preceding simple aspergilloma, while chronic fibrosing aspergillosis is extremely rare [7,37]. Allergic bronchopulmonary aspergillosis is occasionally observed [7]. In the largest study of chronic pulmonary aspergillosis complicating sarcoidosis, patients presented with the following symptoms: cough (86%), hemoptysis (36%), fever (29%), and weight loss (40%) [7]. All but 1 patient (64 out of 65) had at least one cavitation, with multiple cavitations observed on lung CT scans [7]. Positive *Aspergillus* serology was observed in 92% of patients, while *Aspergillus* was found in 77.9% of bronchial endoscopic or sputum specimens. Coinfection with bacteria was observed in 46.4% of patients, mainly with *Pseudomonas aeruginosa*. Nontuberculous mycobacteria were also detected. Serum C-reactive protein levels were elevated in 87% of cases. The severity of pulmonary sarcoidosis was demonstrated by a Composite Physiologic Index score above 40 in 62% of cases, with pulmonary fibrosis present in almost 90% of cases, with an average extent of 22%. Pulmonary hypertension was detected in 30.7% of the cases. Sixty-seven % of patients met the high-risk prognostic criteria according to Walsh [38]. The survival rate was comparable to a control group of sarcoidosis patients, who were matched with patients without Aspergillus infection based on their fibrocystic pattern and the date of fibrocystic lung detection on the imaging. Specifically, the survival was 73% at 5 years and 61% at 10 years. Although 3 patients died due to massive hemoptysis, interventional radiology effectively managed 14 cases of massive hemoptysis. The use of antifungal medications was shown to be effective based on symptom resolution and improvement in the chest CT. In particular, a decrease in the maximum thickness of the cavity wall and pleura has proven to be the most discriminating factor in evaluating the therapeutic response [39]. Nevertheless, the complete response and long-term recovery were rare occurrences, probably due to the persistent cavitary lesions in fibrocystic lung. To date, there are no studies comparing antifungal drugs and their duration of treatment, and thus no protocol can be recommended. However, a recent interesting prospective study on the duration of itraconazole treatment in chronic pulmonary aspergillosis in underlying lung diseases other than sarcoidosis suggested a better outcome at 2 years, with fewer relapses when the treatment duration was extended to 12 months compared to 6 months [40]. High occupational exposure is a risk factor for chronic pulmonary aspergillosis. This condition is associated with jobs that have a high risk of exposure to molds (37.5%) compared to sarcoidosis patients without aspergillosis infection (17.5%) [7].

## 7. Suprainfections in Fibrotic Lung Lesions

Baughman et al. observed the frequent occurrence of bacterial suprainfections responsible for acute exacerbation in fibrotic lung sarcoidosis, with a favorable response to short courses of antibiotics [5]. Bronchiectasis and the use of immunosuppressants, especially TNFα blockers, have been associated with an increased risk of infection [5]. In a monocentric study of 142 patients with fibrosing pulmonary sarcoidosis, Nardi et al. found that chronic pulmonary aspergillosis occurred in 11.3% of cases, while tuberculosis occurred in 7% of cases, both during the 7.1-year follow-up of the study. Nontuberculous mycobacterial infections accounted for 2% of cases, while pneumonia due to various agents accounted for 7%. In this series, 1 out of 16 deaths was attributed to *Nocardia* infection [6]. Although dedicated studies on the subject are lacking, patients with advanced pulmonary sarcoidosis often experienced suprainfections, including those caused by *Pseudomonas aeruginosa*, a common pathogen associated with bronchiectasis.

## 8. Tuberculosis

One-third of the world’s population carries latent tuberculosis infection [16]. The majority of these people live in countries where tuberculosis is endemic. Therefore, clinicians can use the TB profile reference [41] to assess the incidence of tuberculosis in different regions and determine the potential threat of tuberculosis. Some individuals in countries with low incidence of tuberculosis may contract the disease through travel to high-incidence countries or contact with an infectious person. 

Sarcoidosis patients undergoing immunosuppressive therapy, especially with corticosteroids or TNFα blockers, are at increased risk of developing tuberculosis. It is widely recognized that differentiating between pulmonary sarcoidosis and tuberculosis can be challenging due to confusing imaging and even pathology. Granulomas are not always necrotic in tuberculosis, whereas fibrinoid necrosis is possible in sarcoidosis. Microbiologic studies for *M. tuberculosis* may produce false-negative results, even when tuberculosis is present [42]. Diagnosing tuberculosis suprainfection in a patient with confirmed sarcoidosis is a difficult task, with a high risk of overdiagnosing a sarcoidosis exacerbation. Such a misdiagnosis may lead to the initiation or escalation of immunosuppressive treatment, with potentially disastrous consequences. The rule should be to consider an alternative cause—such as an infectious, cardiac, or thromboembolic cause—when there is an unexpected progression of symptoms in a patient who previously had a well-controlled disease and no recent changes in their treatment regimen. In this context, it is important to have a thorough understanding of the patient’s risk factors. If a patient has a history of travel in a highly endemic country, and presents with general symptoms, such as fever or weight loss, productive cough with purulent sputum, hemoptysis, and new chest CT imaging findings, such as cavities or necrotic lymphadenopathy, may suggest mycobacterial infection. Microbiological stains and techniques can be used to look for mycobacteria in sputum, bronchoalveolar lavage, or tissue samples. It is important to note that interferon-γ release assays and tuberculin tests can produce false-negative results, especially in elderly patients with low blood lymphocyte counts. Wang’s research showed a significant increase in the risk (×1.85) for tuberculosis in sarcoidosis patients [16]. However, the study did not compare the risk between patients based on their sarcoidosis treatment. Moreover, the close temporal association between tuberculosis and sarcoidosis diagnosis, coupled with the challenge of distinguishing the two diseases, raises the possibility of the misdiagnosis of sarcoidosis as tuberculosis in some patients [16].

## 9. Coronavirus Disease 2019 (COVID-19) and Sarcoidosis

### 9.1. COVID-19 Severity in Patients with Sarcoidosis?

The 2019 coronavirus pandemic was caused by severe acute respiratory syndrome coronavirus 2 (SARS-CoV-2). Several risk factors for admission in the intensive care unit (ICU) and/or mortality have been identified, including advanced age, male sex, and comorbidities, such as obesity, cardiovascular disease, diabetes mellitus, and chronic respiratory disease [43]. Since 2020, several cohort studies have attempted to determine whether patients with sarcoidosis are at an increased risk of developing severe COVID-19 or experiencing poor outcomes (Table 1). It is important to note that all the studies discussed here regard the severity of enrolled patients at the onset of the pandemic, before the introduction of vaccines and the emergence of new, less severe variants.

Several series studies with small numbers of patients with sarcoidosis and COVID-19 have reported significantly high rates of ICU admission and mortality, reaching 37.5% and 17.2%, respectively [44,45,46,47,48,49]. These series had selection biases, as the research was primarily conducted in hospital-based and tertiary centers, potentially leading to the inclusion of a greater proportion of hospitalized COVID-19 patients and individuals with more severe sarcoidosis who had additional comorbidities and were undergoing immunosuppressive treatments. For example, the study conducted by Chevalier et al. analyzed 1213 patients with autoimmune/inflammatory rheumatic diseases with COVID-19 from two French national databases: the EDS (Entrepôt des Données de Santé, including all patients followed in Paris university hospitals) and the French multicenter COVID-19 cohort French Rheumatic and Musculoskeletal diseases (RMDs). Among the 64 patients with both sarcoidosis and COVID-19, the study found that sarcoidosis was an independent factor for severe COVID-19 (i.e., ICU admission and/or death) in the multivariate analysis (aOR = 5.19, 2.15–12.3), along with other factors, such as older age, interstitial lung disease, arterial hypertension, and obesity [49]. 

In contrast, a representative with a large unselected cohort in England was designed to provide a risk estimate for severe COVID-19 among individuals with chronic respiratory disease, while adjusting for demographic and socioeconomic status and comorbidities associated with severe COVID-19 [50]. This study used a database from English general practices, which was linked to Public Health England’s database of SARS-CoV-2 testing, and records of English hospital admissions, intensive care unit (ICU) admissions, and COVID-19-related deaths [50]. The cohort for this study consisted of 8,256,161 individuals, of whom 14,479 (0.2%) were hospitalized with COVID-19 and 5956 (0.1%) died. Among the 17,624 patients with sarcoidosis, 84 (0.5%) required hospitalization and 32 (0.2%) succumbed to the disease. The study found that individuals with sarcoidosis, along with other respiratory diseases, like COPD and ILD, had an increased risk of hospitalization, but not ICU admission, with a hazard ratio of 1.36 (1.10–1.68), compared to those without these respiratory diseases after adjusting for comorbidities. Patients with sarcoidosis were also at increased risk of death, but with imprecise estimates (HR 1.41 (0.99–1.99)). According to the authors, sarcoidosis patients appeared to have a modestly increased risk of severe disease, but their risk of death from COVID-19 at the height of the epidemic was mostly much lower than the usual risk of death from any cause [50]. 

Another large retrospective cohort study in the United States examined the risks of COVID-19 in patients with pulmonary sarcoidosis compared to a propensity-matched cohort on comorbidities and the demographics of patients without sarcoidosis using a multicenter research network called TriNEXT. The study identified a total of 278,271 COVID-19 patients within the research network, of which 954 patients (0.34%) had a diagnosis of pulmonary sarcoidosis. Common comorbidities, including hypertension, chronic lower respiratory disease, diabetes mellitus, ischemic heart disease, nicotine dependence, and chronic kidney disease, were found to be more prevalent in patients with pulmonary sarcoidosis. In the initial unmatched analysis, the pulmonary sarcoidosis group had a higher mortality rate (4.3% versus 2.06%) and an increased risk of ICU admission (6.92% versus 3.05%). However, after applying propensity score matching, no significant differences were observed between the groups [51]. These analyses suggest that the higher mortality observed in sarcoidosis patients may be due to the increased burden of comorbidities rather than the disease itself.

A smaller nationwide retrospective study conducted in Spain showed similar results in assessing the clinical status of patients with systemic autoimmune diseases (SADs) hospitalized with COVID-19. The study included 149 sarcoidosis patients. It was found that the in-hospital mortality was higher in patients with SADs compared to the control group (20% vs. 16%, *p* < 0.001). However, after adjustment for baseline conditions, SADs were not associated with a higher risk of mortality (odds ratio = 0.93; 95% confidence interval, 0.78–1.11). The mortality observed in patients with SADs was mainly influenced by factors such as age, heart failure, chronic kidney disease, and liver disease [52].

The risk of severe COVID-19 in sarcoidosis patients seems to be mainly influenced by comorbidities. However, it is conceivable that the severity may be partly due to the impaired lung function associated with sarcoidosis or the use of immunosuppressive drugs [45,46].

Morgenthau et al. found that sarcoidosis was associated with severe COVID-19 outcomes only in patients with moderately and/or severely impaired pulmonary function (aOR 7.8; 95% CI, 2.4–25.8), independent of demographics and comorbidities [46].

Immunosuppressive medications may be associated with the risk of severe sarcoidosis. In the study by Chevalier et al., treatment with corticosteroids (aOR 2.47 (1.58–3.87)) or rituximab (aOR 3.32 (1.45–7.49)) was an independent factor of severe COVID-19 [49]. It seems that the risk only affects patients with a corticosteroid dose ≥10 mg/day [53]. Interestingly, treatment with leflunomide and methotrexate was significantly associated with a better outcome [49]. The TNF-alpha antagonists do not seem to be associated with severe COVID-19 in this and other studies [44,49,53]. The effect of corticosteroids on severe COVID-19 in sarcoidosis and other immune system diseases may be driven by a defective ability to respond to the vaccine and/or to control SARS-CoV-2 infection. In a small series of sarcoidosis patients, corticosteroids were associated with a defective T-cell response against the spike protein [54].

In conclusion, the risk of severe COVID-19 (i.e., ICU admission and/or death) associated with sarcoidosis appears to be moderate, especially in large unselected studies. However, the increased severity observed in these patients may be driven by known comorbidities associated with COVID-19 (e.g., age, cardiovascular disease, etc.), severe ILD, and the use of glucocorticoids. As the studies were conducted before the advent of vaccines, it is possible that the risk of severe COVID-19 associated with sarcoidosis is even lower now.

### 9.2. Risk of Sarcoidosis and Sarcoidosis Flare-Ups after COVID-19 and LINKS to Pathogeny

Several case reports and series have reported the detection of sarcoidosis following COVID-19 [55,56]. Rare cases of sarcoidosis flares have also been reported in a few isolated case reports [57,58]. These flare-ups can be severe, as evidenced by the description of three patients who presented with cardiac sarcoidosis and ventricular tachycardia following SARS-CoV-2 infection [58]. Certainly, further research is warranted to understand how inflammatory processes during COVID-19 might trigger or intensify sarcoidosis activity. While the overall risk of infection-triggered sarcoidosis appears to be remarkably low among the large population of patients with a history of COVID-19, this suggests that various risk factors may contribute to the development of this disease in the relatively few cases reported to date [4]. A recent retrospective population-based study using nationwide data in Korea found that individuals with COVID-19 had a significantly increased risk of developing sarcoidosis (aHR, 1.59; 95% CI, 1–2.52). However, it is important to note that the confidence interval in this study was imprecise and the number of incident sarcoidosis cases per year in the COVID-19 group was low (*n* = 3) [59].

Sarcoidosis and COVID-19 may share some common mechanistic immune responses, including the renin–angiotensin system in the lungs and some cell death pathways related to the regulation of autophagy [60], apoptosis, and programmed cell death (PD-1/PD-L1 axis) [61]. 

Pacheco et al. conducted a study aimed at identifying the genetic factors that could potentially increase the susceptibility of sarcoidosis patients to severe forms of SARS-CoV-2 infection. Their research involved a comprehensive whole-exome screening of 13 predisposed to sarcoidosis families and a healthy control group. The team then compared the genes sharing mutations with the list of genes involved in the SARS-CoV-2 host–pathogen protein–protein interactome. 

Their results showed that approximately 10% of the genes listed in the SARS-CoV-2 interactome were affected by pathogenic mutations shared between sarcoidosis patients and controls. These mutations were found to disrupt interactions between host and viral proteins during infection. In particular, the RIG-I (retinoic acid-inducible gene 1)/MDA-5 pathway was identified as the primary affected pathway, leading to the attenuation of antiviral immunity and the facilitation of viral replication.

In addition, sarcoidosis patients were found to accumulate a significant number of mutations in genes associated with intracellular trafficking and the regulation of autophagy and mitophagy, with a particular focus on the mTOR functional hub. The researchers postulated that sarcoid granulomas may potentially represent the pathogenic manifestation of a common response to various environmental triggers and viral infections [62].

## 10. Treatments

### 10.1. Curative Treatments

Two points need to be considered: first, to provide appropriate anti-infective treatment; second, the reconsideration of immunosuppressive treatment depending on the stakes involved. To achieve this goal, the best means to isolate the responsible infectious agent should be used. There are no specific guidelines for the treatment of respective infections in the specific situation of sarcoidosis patients. Thus, in most situations, anti-infective treatments will be administered according to the guidelines developed for other diseases, when available. 

There are insufficient data concerning how to modify immunosuppressive treatments in case of suprainfection. In addition, the situation may vary greatly depending on the infectious agent, the efficacy of available anti-infectious drugs, and the severity and control of sarcoidosis. In the case of severe infection, most authors tend to stop or reduce immunosuppressive drugs with not-unfrequent ulterior relapses of sarcoidosis [24,34]. This is particularly indicated when the efficacy of the anti-infective treatment is uncertain, as in PML.

### 10.2. Preventive Measures

Preventive measures for tuberculosis include hygienic practices, prophylactic medications, and vaccines.

#### 10.2.1. Hygiene Measures

Hygiene measures should be explained to patients receiving immunosuppressive treatment, especially for those traveling to areas with a high incidence of tuberculosis (with the recommendation to wear a protective face mask). Although research on this topic is still limited, it is advisable to inform patients with advanced pulmonary sarcoidosis about the dangers associated with heavy exposure to molds in the workplace and at home.

#### 10.2.2. Preventive Medications

Preventive treatments may be used to reduce the risk of pneumocystosis, herpes zoster, and tuberculosis. Although sarcoidosis is considered a low risk for *Pneumocystis* infection, authors recommend the use of prophylactic antimicrobials [4,26]. Specific guidelines for immunosuppressed patients should be implemented in treated sarcoidosis patients [63,64]. The identification of possible sources of transmission is essential. The use of tuberculin skin test and/or an interferon-gamma release assay is recommended, as well as a systematic treatment involving isoniazid or rifampicin for confirmed latent tuberculosis [63,64]. Interestingly, antimycobacterial therapy does not benefit sarcoidosis patients without a tuberculosis association, as confirmed by the CLEAR study [65]. 

#### 10.2.3. Vaccination and Sarcoidosis

##### Is It Safe? What Is the Efficacy?

In sarcoidosis, inflammation is thought to result from maladaptive immune responses triggered by chronic immune stimulation, leading to an increased risk of lymphocyte anergy, exhaustion, and depletion [66,67]. This impaired immune response could potentially result in a reduced vaccine efficacy compared to the general population. Current data on the efficacy of vaccination in sarcoidosis patients are limited and conflicting. For example, in a study focusing on tetanus vaccination in 48 sarcoidosis patients, it was found that 50% of the participants had an inadequate increase in antibody titers, regardless of their sarcoidosis disease status, stage, duration, or ongoing treatment [68]. Another study, evaluating a three-dose series of the hepatitis B vaccine, showed that none of the 16 subjects with sarcoidosis had detectable antibody levels during the 1-month follow-up period [69]. In contrast, a study focusing on the trivalent influenza vaccine showed that both subjects with sarcoidosis (*n* = 23) and controls had a similar serologic response [70]. Recently, a study testing the efficacy and response on the COVID-19 vaccine showed that 14 subjects with sarcoidosis had a decreased quantitative antibody (antitrimer) response to the BNT162b2 mRNA COVID-19 vaccine, but their functional neutralizing antibody response was comparable to controls, indicating conferred immunity. Their results suggest that sarcoidosis subjects mount a robust initial trimer IgG antibody response to vaccination, with a subsequent quantitative decline by 6 months, perhaps driven by those on immunosuppression [71].

Certainly, the use of immunosuppressive drugs has been associated with decreased antibody responses to various types of vaccinations, including mRNA COVID vaccines. However, data on the effect of immunosuppressive drugs on the vaccine efficacy on sarcoidosis are limited, and recommendations are often inferred from studies of other immune-related diseases [72,73,74]. For example, if the disease activity permits, methotrexate is recommended for 2 weeks after the influenza vaccination, pneumococcal vaccination [72], and COVID-19 booster vaccine [75]. A meta-analysis of 13 studies involving 886 rheumatoid arthritis patients evaluated the rates of seroprotection, which were similar between rheumatoid arthritis patients on glucocorticoids and healthy controls [76]. 

Vaccination is widely considered to be a safe, effective, and cost-effective measure that can potentially reduce the morbidity and mortality associated with sarcoidosis patients [77]. The current literature on sarcoidosis is too limited to clearly state whether or not vaccination exacerbates or induces sarcoidosis. However, some authors have hypothesized a role for the adjuvant in inducing inflammatory, autoimmune diseases, and sarcoidosis [78,79,80]. In a small study of influenza vaccination in sarcoidosis patients, no evidence of disease flares or serious adverse events were observed in the sarcoidosis group after 6 months of follow-up [70]. More recently, a nationwide population-based study was conducted in South Korea to investigate the incidence and risk of autoimmune connective tissue diseases after the mRNA-based COVID-19 vaccination. Among 3,838,120 vaccinated individuals, the study found no increased risk of developing sarcoidosis or other autoimmune disorders compared to unvaccinated controls. However, caution should be exercised in interpreting the results for rare outcomes due to the limited statistical power of the study [81]. Interestingly, in a Danish-registry-based incidence study, the period of high BCG vaccination uptake was associated with a lower incidence rate of sarcoidosis, mostly in men. This supports the hypothesis of a potential protective effect of BCG vaccination against the development of sarcoidosis, which could be due to a trained immunity against *Mycobacterium* spp. [82]. 

##### When Is It Indicated?

Given the limited data available on the efficacy of vaccination for sarcoidosis, Syed et al. used evidence from the vaccination of immunosuppressed populations to propose general vaccination recommendations for sarcoidosis patients. These recommendations have been endorsed by the World Association of Sarcoidosis and Other Granulomatous Disorders [83]. Essentially, their recommendation was to administer inactivated vaccines, including pneumococcal, influenza, and hepatitis B vaccines, regardless of the patient’s current immunosuppressive regimen. Live attenuated vaccines should be administered prior to the initiation of any biologic therapy and should be avoided if the patient is already on a biologic therapy. Of note, these recommendations have been endorsed by the World Association of Sarcoidosis and other Granulomatous Disorders [77]. 

Of note, a population-based study in Sweden found that the pneumococcal conjugate vaccine administered during the childhood immunization program was associated with an increased burden of nonvaccine serotypes of invasive pneumococcal disease in individuals with comorbidities, including those with sarcoidosis. This finding suggests the potential need for the administration of the 23-valent pneumococcal polysaccharide vaccine (PPV23) in this population [84]. 

Given the severity of the COVID-19 pandemic, and the increased risk of severe pulmonary outcomes in sarcoidosis, experts in sarcoidosis strongly recommend that patients with sarcoidosis receive the COVID-19 vaccination [83], especially those with comorbidities, impaired pulmonary function, and those taking immunosuppressive drugs.

## 11. Are Anti-Infectious Mechanisms Associated with Sarcoidosis Impaired?

As developed above, and already mentioned [4], the relationship between sarcoidosis and infection is complex and difficult to decipher. The first question would be to consider sarcoidosis per se as a fertile ground for bacterial, fungal, or viral infections, but infections are relatively rare. The second question would be to consider infections as a consequence of tissue damage related to sarcoidosis, such as pulmonary fibrotic and destructive lesions, or immunodepression related to the treatments patients receive (steroids, immunosuppressants, etc.). Furthermore, this topic is particularly complex because infectious agents are suspected to be causative agents in sarcoidosis. 

Sarcoidosis is characterized by a paradoxical immune status, i.e., an exaggerated immune response within the granulomas, in contrast to various immune defects, as indicated by the anergy to the tuberculin test and the occurrence of some opportunistic infections [85]. However, to the best of our knowledge, studies of immunity in sarcoidosis have focused primarily on the pathogenesis of the disease, and studies on the possible impairment of the anti-infectious response are rare, with the exception of vaccination. When considering humoral immunity, the different steps of its knowledge can be summarized as follows. Fifty years ago, a lymphopenia observed mainly in T-lymphocytes, but not in B-lymphocytes, was demonstrated in sarcoidosis patients [86]. Subsequently, suppressor cells, at that time monocytes, were experimentally suspected to be responsible for the immunological abnormalities [87]. Later, T-activated lymphocytes were shown to be active in controlling antibody production, and thus modulating the polyclonal hyperglobulinemia observed in sarcoidosis [88]. Later, a disturbance in B-cell differentiation was observed, with an in vitro decrease in the production of IgG1, IgG3, and IG subclasses [89]. However, in contrast to patients with common variable immunodeficiency, normal levels of total serum IgG, IgA, and IgM, as well as IgG and IgA subclasses, were observed in a series of 32 patients [90]. In addition, patients in this series had normal vaccination responses to the influenza virus (seasonal influenza and Mexican influenza) and encapsulated bacteria (Streptococcus pneumoniae), with normal antigen-specific immunoglobulin responses, whereas the B-memory cells were reduced. More recently, emphasis has been placed on the disruption of Th1, Th17, and Treg lymphocytes. In a comparative series of sarcoidosis and autoimmune diseases, a high level of regulatory T-helper cells > 5.70% was observed in the blood of 91% of sarcoidosis patients [91]. Tregs interacting with innate and adaptive immunity have been shown to limit acute lung inflammation, due to respiratory pathogens, and to provide lung protection [92]. A decrease in the absolute number of circulating Tregs and several alterations in Treg cell subsets have been reported in sarcoidosis [93], and more recently, the cross-talk of B-cells with regulatory T-follicular helper cells (Tfh) has been shown in sarcoidosis [94], suggesting that Tfh2- and Tfh17-like cells—the most effective cell type in supporting B-cell activity, particularly in antibody production—may play a role in the anti-infectious humoral response in sarcoidosis. In conclusion, although the references are not extensive on this topic, the humoral arm does not seem to be defective in sarcoidosis, except against still-unknown very-selective targets, explaining the stochastic occurrence of infections in sarcoidosis.

Two fungi, *Cryptococcus* and *Aspergillus*, are associated with opportunistic infections in sarcoidosis. Macrophages are essential to control mycoses due to *Cryptococcus*, while neutrophils are critical against *Aspergillus* [95]. 

Cryptococcosis, while rare, is significantly associated with sarcoidosis [30]. The impairment of cell-mediated immunity and long-term corticosteroid therapy is being evoked to explain this association. But, as reported in the CryptOsarc study, cryptococcosis occurred in one-third of the cases in patients without any treatment [30]. In this study, as well as that reported by Prevel et al., peripheral blood CD4 lymphocytopenia was not an independent risk factor [30,85]. The alteration of qualitative CD4 T-cell function could be involved in the pathophysiology, but T-cell dysfunction in sarcoidosis is poorly understood [85,96]. An altered CD4 T-cell–macrophage crosstalk has experimentally been demonstrated to be involved via the decreased macrophage ability to contain *Cryptococcus* spp. [97]. For instance, macrophage-like cells, called B-1-derived mononuclear phagocytes (BDMPs), have demonstrated to phagocyte *Cryptococcus neoformans* via a complement receptor 3-mediated pathway. This BDMP cell could be one key in the defense against *Cryptococcus*, but it is largely speculative [85]. 

Anti-GM-CSF antibodies were found in a subset of patients with sarcoidosis, which may impair macrophage phagocytic function and may be another additional mechanism [85].

Aspergillus, suspected to be a driver of sarcoidosis [98], causes chronic pulmonary aspergillosis, which complicates sarcoidosis, with fibrocystic lung remodeling [7]. The pathogenesis of aspergilloma usually involves the colonization and proliferation of the fungus in a pre-existing lung cavity [95]. Neutrophils play a key role in the defense against Aspergillus through phagocytosis, oxidative bursts, and the formation of neutrophil extracellular traps (NETs). This process has received considerable attention and has made rapid progress since NETs [99]. However, to the best of our knowledge, no alterations in neutrophil function have been described in sarcoidosis patients. PML is caused by the human polyomavirus 2/JCV, and is usually associated with immunodeficiency. It can be observed without the overt immunosuppression [100] reported in neurosarcoidosis in immunocompetent adults [101]. Peripheral CD4 lymphocytopenia, evoked by lymphocytic sequestration in granulomas and peripheral anergy, have been discussed, but no clear mechanism of virus escape from immune vigilance is yet proposed.

Finally, it can be hypothesized that one mechanism favoring infections could be the impairment of the autophagy machinery reported in sarcoidosis [62,102]. Autophagy has been implicated in intercepting microbes using various receptors, such as TLR- and NOD-dependent detection for bacteria [103]; however, no association between NOD mutations and an increase in bacterial infections has been reported. NOD2 has an important role in mycobacterial recognition, but the mechanisms by which NOD2 mutations are involved in mycobacterial infection are still unclear [104]. Upon viral infection, autophagy could fight invading viruses by degrading viral particles, initiating the innate immune response, and facilitating viral antigen presentation, all of which contribute to the prevention of viral infection and pathogenesis [105]. However, autophagy and its mechanisms are so complex that it is very difficult to decipher its role in the very rare infectious events associated with sarcoidosis. 

## 12. Discussion

First, we would like to provide some take-home messages derived from the relevant sections of this article: (i) to the best of our knowledge, no active infectious disease can be considered as a cause of sarcoidosis, not even tuberculosis; (ii) immunosuppressive drugs given to treat sarcoidosis and fibrocystic lung lesions in advanced pulmonary sarcoidosis may increase the risk of various infections; (iii) latent tuberculosis must be investigated and treated; (iv) infectious diseases must not be overlooked and must be systematically considered in cases of unclear worsening or new localization; (v) immunosuppressants must be optimally adjusted without excess in dose or duration; (vi) patients should receive inactivated vaccines, including pneumococcal, influenza, hepatitis B, and COVID-19 vaccines, regardless of the patient’s current immunosuppressive regimen. Regarding the high risk of sarcoidosis following tuberculosis in Wang’s study, it is important to emphasize the potential bias arising from the similarities between the presentations of sarcoidosis and tuberculosis [16]. Despite an interesting study on the noninfectious adverse events of corticosteroids in sarcoidosis [106], the risk of infectious diseases associated with immunosuppressants has not been thoroughly investigated in well-designed studies, especially with regard to the duration of surveillance. This gap exists despite well-conducted studies in sarcoidosis trials [107], and even in rheumatoid arthritis trials [22]. However, thanks to observational studies, the risks have been well-identified.

It is important not to overlook infectious events during follow-up. The main differential diagnosis is sarcoidosis progression, and for the lungs, heart failure or pulmonary thromboembolism. The diagnosis of sarcoidosis progression is mainly based on the serial assessment of symptoms, pulmonary function tests, and imaging [108,109]. For new extrapulmonary manifestations, the use of the WASOG sarcoidosis organ instrument allows for the reduction in the overdiagnosis of sarcoidosis sites [110]. Any atypical finding, especially an unexpected worsening, for example, in a recently well-controlled disease with stable treatment, needs to be investigated. Epidemiologic information, such as travel to a country with a high tuberculosis endemicity, is also essential. In these cases, a multidisciplinary discussion may be helpful.

Some points are still under discussion. There is a need to determine which antifungal drug to prioritize and whether prophylaxis against pneumocystosis should be systematically given to treated patients.

## 13. Future Research Direction

Key information is lacking, particularly regarding the infectious risks of using corticosteroid-sparing agents. Infectious risks need to be carefully assessed in trials. They can be included as secondary outcomes and studied over several years. For certain drugs, registries may also be helpful. The rarity of events, which is unusual even for rare diseases, poses a challenge to the conduct of trials. Designated trials may provide more insight. The pathogenesis of opportunistic infections, such as PML and cryptococcosis, in untreated sarcoidosis requires dedicated research.

## 14. Conclusions

Infectious diseases can occur in the course of sarcoidosis, mainly due to the use of immunosuppressants, advanced pulmonary lesions, and various epidemiologic risks associated with tuberculosis, certain fungal infections, or COVID-19. Early recognition and understanding of these infections are critical, even though their diagnosis may be obscured by sarcoidosis-related findings. Preventive measures are also important. There is a particular need for studies comparing the risk of infection between different corticosteroid-sparing treatment protocols.

## Figures and Tables

**Table 1:** Summary of the studies evaluating the risk of severe COVID-19 associated with sarcoidosis.

Population Study with COVID-19 (*n*)	Patients with COVID-19 and Sarcoidosis (*n*)	Hospitalizations *n* (%)	ICU Admision *n* (%)	Deaths *n* (%)	Mode of Recruitment/Location	Risk of Severe COVID-19 Associated with Sarcoidosis	Ref.
36	36	28 (78%)	13 (36%)	5 (14%)	Tertiary centers/France	NA	[44]
45	45	14 (31%)	2 (5%)	4 (9%)	Nationwide, multicenter registry from tertiary centers/Spain	NA	[45]
7337	37	22 (59.5%)	NA	6 (16.2%)	Tertiary centers/US	Not associated with intubation and mechanical ventilation or in-hospital mortality	[46]
119	77	19 (24.7%)	6 (7.9%)	1 (1.3%)	One tertiary center/US	NA	[47]
8	8	3	2	1	One tertiary center/France	NA	[48]
1213	64	NA	24 (37%)	11 (17%)	Paris University Hospital Database and a national cohort from tertiary centers/France	Independent factor of severe COVID-19 (i.e., hospitalization in the ICU and/or death) (aOR = 5.19, 2.15–12.3)	[49]
8,256,161	17 624	84 (0.5%)	10 (0.1%)	32 (0.2%)	Database from English general practices linked to Public Health England’s database of SARS-CoV-2 testing, along with records of English hospital admissions, ICU admissions, and COVID-19-related deaths	Independent factor (after adjusting for comorbidities) of hospitalization (but not in ICU, HR 1.36 (1.10–1.68)), and of increased death HR 1.41 (0.99–1.99)	[50]
278,271	954	185 (19.4%)	66 (6.9%)	41 (4.31%)	Multicenter research network TriNETX in United States. Participating organizations: large academic centers that operate both tertiary care and satellite secondary or primary office locations	No significant association between sarcoidosis and the risk of hospitalization, nor a higher morality, when adjusted for demographics and comorbidities	[51]
117,694	892 (systemic autoimmune diseases) 149 (sarcoidosis)	All hospitalized	98 (11%)	174 (20%)	Nationwide retrospective study Spain using ICD10 codes in the National Registry of Hospital Discharges	SADs not associated with a higher risk of COVID-19 mortality (OR = 0.93; 95% CI, 0.78–1.11)	[52]

## Data Availability

Not applicable.
